# *PLIN1* Haploinsufficiency Causes a Favorable Metabolic Profile

**DOI:** 10.1210/clinem/dgac104

**Published:** 2022-03-02

**Authors:** Kashyap A Patel, Shivang Burman, Thomas W Laver, Andrew T Hattersley, Timothy M Frayling, Michael N Weedon

**Affiliations:** University of Exeter Medical School, Exeter, EX2 5DW, UK

**Keywords:** PLIN1, lipodystrophy, HDL, triglycerides

## Abstract

**Context:**

*PLIN1* encodes perilipin-1, which coats lipid droplets in adipocytes and is involved in droplet formation, triglyceride storage, and lipolysis. Rare *PLIN1* frameshift variants that extend the translated protein have been described to cause lipodystrophy.

**Objective:**

This work aimed to test whether *PLIN1* protein-truncating variants (PTVs) cause lipodystrophy in a large population-based cohort.

**Methods:**

We identified individuals with *PLIN1* PTVs in individuals with exome data in the UK Biobank. We performed gene-burden testing for individuals with *PLIN1* PTVs. We replicated the associations using data from the T2D Knowledge portal. We performed a phenome-wide association study using publicly available association statistics. A total of 362 791 individuals in the UK Biobank, a population-based cohort, and 43 125 individuals in the T2D Knowledge portal, a type 2 diabetes (T2D) case-control study, were included in the analyses. Main outcome measures included 22 diseases and traits relevant to lipodystrophy.

**Results:**

The 735 individuals with *PLIN1* PTVs had a favorable metabolic profile. These individuals had increased high-density lipoprotein cholesterol (0.12 mmol/L; 95% CI, 0.09 to 0.14, *P* = 2 × 10^–18^), reduced triglycerides (–0.22 mmol/L; 95% CI, –0.29 to –0.14, *P* = 3 × 10^–11^), reduced waist-to-hip ratio (–0.02; 95% CI, –0.02 to –0.01, *P* = 9 × 10^–12^), and reduced systolic blood pressure (–1.67 mm Hg; 95% CI, –3.25 to –0.09, *P* = .05). These associations were consistent in the smaller T2D Knowledge portal cohort. In the UK Biobank, *PLIN1* PTVs were associated with reduced risk of myocardial infarction (odds ratio [OR] = 0.59; 95% CI, 0.35 to 0.93, *P* = .02) and hypertension (OR = 0.85; 95% CI, 0.73 to 0.98, *P* = .03), but not T2D (OR = 0.99; 95% CI, 0.63-1.51, *P* = .99).

**Conclusion:**

Our study suggests that *PLIN1* haploinsufficiency causes a favorable metabolic profile and may protect against cardiovascular disease.

*PLIN1* encodes perilipin-1, which coats lipid droplets in adipocytes and is involved in droplet formation, triglyceride storage, and lipolysis. A candidate gene approach in family-based studies showed that rare heterozygous *PLIN1* frameshift variants cause severe monogenic partial lipodystrophy (1-3). Lipodystrophies are a group of disorders that are characterized by varying degree of subcutaneous fat loss leading to insulin resistance, diabetes, severe dyslipidemia, hepatic steatosis, and significant predisposition to atherosclerotic cardiovascular disease.

Haploinsufficiency was originally proposed as a mechanism explaining the autosomal dominant phenotype of *PLIN1*-related lipodystrophy (1). However, we studied 6 individuals with protein-truncating variants (PTVs) of *PLIN1* who lacked features of lipodystrophy and had normal lipid profiles (4). Our work suggested haploinsufficiency could not be the mechanism leading to lipodystrophy. Instead, only specific frameshift variants that produce an extended PLIN1 protein with an alternate C-terminus may cause the disease (2). *PLIN1* studies have so far been based on a small number of clinically ascertained individuals, so there is still a question as to the role of *PLIN1* PTVs in lipodystrophy (2).

Recently the UK Biobank released exome data on 454 787 individuals with detailed clinical and biomarker data available (5). This provides a unique opportunity to test the effect of *PLIN1* variants on lipodystrophy phenotypes in a population-based setting. A genotype-first approach provides a better estimate of the effect of these variants because carriers are not subject to the ascertainment biases of clinical presentation. Here we show that, rather than causing a lipodystrophy phenotype, *PLIN1* haploinsufficiency causes a favorable metabolic profile and may protect against cardiovascular disease. We suggest that novel PTVs in *PLIN1* should not be reported as a cause of lipodystrophy.

## Material and Methods

### Phenotypes

We focused our analyses on traits and diseases that had been previously reported to be associated with *PLIN1* frameshift mutations and that were available in the UK Biobank. The UK Biobank is a population-based cohort from the United Kingdom with deep phenotyping data and genetic data for around 500 000 individuals aged 40 to 70 years at recruitment (6). Participants provided a range of information via questionnaires and interviews including self-report disease status. In addition, a panel of biomarkers was measured from blood and urine. Phenotypes were derived from medical history interviews, inpatient and outpatient International Classification of Diseases, Ninth Revision (ICD-9) and Tenth Revision (ICD-10) codes, operation codes, and death registry data. Supplementary Table 1 (7) provides details of how each individual phenotype used in this study was generated for continuous traits. Supplementary Table 2 (7) provides all the self-report, ICD-10, ICD-9, OPCS (Office of Population Censuses and Surveys), and other codes used to define disease status in the UK Biobank analyses. We used all incident and prevalent cases for each of the diseases. For type 2 diabetes (T2D) we used a strict definition excluding any individual with diabetes who reported using insulin within 1 year of diagnosis, were diagnosed before age 35 years, or were diagnosed within the past year. The UK Biobank resource was approved by the UK Biobank Research Ethics Committee and all participants provided written informed consent to participate. We included only unrelated European ancestry individuals in this analysis as defined in Jones et al (8).

### Exome Sequencing

A subset of approximately 450 000 DNA samples from UK Biobank participants underwent exome sequencing; this data set was recently made available for research and the details, including sequencing technologies, bioinformatics pipeline, and quality control, have been described elsewhere (5). Only variants covered by more than 10 reads for more than 90% of samples were included in the analysis. We manually inspected a random selection of integrated genome viewer plots from each of the variants to assess quality.

### Definition of Protein-truncating Variants

We annotated *PLIN1* variants using AlaMut batch software v1.8 (Interactive Biosoftware) against transcript NM_002666.5. We defined a PTV as a variant annotated as frameshift, nonsense, or an essential splice site. We additionally used Loss of Function Transcript Effect Estimator (LoFTEE) (9) to annotate high-confidence loss-of-function variants, and our main analyses are based on those annotated as high confidence by LoFTEE.

### Type 2 Diabetes Knowledge Portal and Phenome-wide Association Study

The Type 2 Diabetes Knowledge portal (10). The Regeneron burden testing analyses are described in (5). We present only the results for the M1 all-variant mask, which includes all loss-of-function variants in *PLIN1*.

### Statistical Analyses

We performed all analyses in Stata 16. We tested 16 continuous traits and 6 binary traits; a *P* value less than .002 is considered statistically significant at a Bonferroni threshold. All traits were run as the raw trait and, as a sensitivity analysis, as inverse normalized traits. The continuous analyses were performed using linear regression, adjusting for age, sex, center of recruitment, and 5 principal components of ancestry. The binary analyses were performed using a Fisher exact test without adjustments (because of small numbers) and a logistic regression analysis adjusted for age, sex, center of recruitment, and 5 principal components of ancestry.

## Results

### *PLIN1* PTVs are associated with a favorable metabolic profile, including reduced waist-to-hip ratio, blood pressure, and triglycerides and increased high-density lipoprotein (HDL) and APOA1

There were 41 *PLIN1* PTVs classified as high confidence by LoFTEE (9) present in 735 individuals in the UK Biobank. Details of these PTVs including their frequency (range, 1-185 individuals) in the UK Biobank are presented in Table 1, and include 21 frameshifts, 15 nonsense, and 5 essential splice-site variants. Table 2 presents gene burden test results for *PLIN1* PTVs in lipodystrophy-related traits available in the UK Biobank. Individuals with *PLIN1* PTVs had increased HDL cholesterol (0.12 mmol/L; 95% CI, 0.09 to 0.14, *P* = 2 × 10^–18^), increased APOA1 (0.07 g/L; 95% CI, 0.05 to 0.09, *P* = 5 × 10^–12^), reduced triglycerides (–0.22 mmol/L; 95% CI, –0.29 to –0.14, *P* = 3 × 10^–11^), and reduced diastolic (–0.87 mm Hg; 95% CI, –1.81 to –0.08, *P* = .06) and systolic blood pressure (–1.67 mm Hg; 95% CI, –3.25 to –0.09, *P* = .05), and reduced waist-to-hip ratio (–0.02; 95% CI, –0.02 to –0.01, *P* = 5 × 10^–12^).

**Table 1.** Details of protein-truncating variants included in this study. Annotated against transcript NM_002666.5

cNomen	pNomen	Coding effect	Exon	UKB No. of carriers (total N = 362 791)
c.46-4_50del		Splice acceptor variant	2	13
c.77del	p.Gln26ArgfsTer5	Frameshift variant	3	2
c.160_166dup	p.Asn56SerfsTer6	Frameshift variant	3	5
c.226del	p.Val76TrpfsTer41	Frameshift variant	3	1
c.247C>T	p.Gln83Ter	Stop gain	3	1
c.251-2A>C		Splice acceptor variant	3	1
c.255dup	p.Ala86SerfsTer4	Frameshift variant	4	1
c.277C>T	p.Arg93Ter	Stop gain	4	79
c.316C>T	p.Gln106Ter	Stop gain	4	1
c.321C>A	p.Tyr107Ter	Stop gain	4	3
c.326del	p.Pro109LeufsTer8	Frameshift variant	4	11
c.326dup	p.Glu110Ter	Frameshift variant	4	2
c.335_350del	p.Ile112ArgfsTer103	Frameshift variant	5	11
c.466del	p.Val156TrpfsTer64	Frameshift variant	5	1
c.502C>T	p.Arg168Ter	Stop gain	5	14
c.511C>T	p.Arg171Ter	Stop gain	6	4
c.589_592del	p.Glu197SerfsTer22	Frameshift variant	6	1
c.602del	p.Pro201LeufsTer19	Frameshift variant	6	2
c.691C>T	p.Arg231Ter	Stop gain	6	2
c.738del	p.Val247TrpfsTer11	Frameshift variant	6	12
c.769dup	p.Leu257ProfsTer40	Frameshift variant	6	1
c.771+1G>A		Splice donor variant	6	75
c.788G>A	p.Trp263Ter	Stop gain	7	1
c.789G>A	p.Trp263Ter	Stop gain	7	9
c.808C>T	p.Gln270Ter	Stop gain	7	1
c.880del	p.Asp294IlefsTer29	Frameshift variant	7	1
c.908del	p.Gly303GlufsTer20	Frameshift variant	7	38
c.964-1G>A		Splice acceptor variant	7	185
c.985C>T	p.Arg329Ter	Stop gain	8	4
c.1012_1022del	p.Thr338AspfsTer51	Frameshift variant	8	197
c.1056G>A	p.Trp352Ter	Stop gain	8	16
c.1057dup	p.Ala353GlyfsTer40	Frameshift variant	8	7
c.1112dup	p.Ala372CysfsTer21	Frameshift variant	8	15
c.1209+1G>A		Splice donor variant	8	1
c.1211_1212insAA	p.Pro405ThrfsTer6	Frameshift variant	9	1
c.1279G>T	p.Glu427Ter	Stop gain	9	1
c.1351C>T	p.Gln451Ter	Stop gain	9	2
c.1398del	p.Gly467AlafsTer74	Frameshift variant	9	1
c.1398dup	p.Gly467ArgfsTer99	Frameshift variant	9	3
c.1401_1405dup	p.Gly469AlafsTer74	Frameshift variant	9	8
c.1544dup	p.Tyr515Ter	Stop gain	9	1

Abbreviation: UKB, UK Biobank.

**Table 2.** Association of protein-truncating variants in *PLIN1* with lipodystrophy-related traits in the UK Biobank

Trait	No. of PTV carriers	PTV carriers mean (SE)	No. of controls	Controls mean (SE)	β (95% CI)	*P*
BMI	727	27.70 (0.19)	361 386	27.36 (0.008)	0.33 (–0.01 to 0.68)	.12
Waist-hip ratio	728	0.85 (0.003)	362 109	0.87 (0.0001)	–0.02 (–0.02 to –0.01)	9 × 10^–12^
HDL cholesterol, mmol/L	642	1.57 (0.02)	317 067	1.45 (0.001)	0.12 (0.09 to 0.14)	2 × 10^–18^
LDL cholesterol, mmol/L	695	3.67 (0.03)	345 569	3.78 (0.002)	–0.11 (–0.17 to –0.05)	5 × 10^–4^
Triglycerides, mmol/L	694	1.53 (0.03)	345 942	1.75 (0.002)	–0.22 (–0.29 to –0.14)	3 × 10^–11^
Apolipoprotein A1, g/L	636	1.61 (0.01)	315 294	1.54 (0.0005)	0.07 (0.05 to 0.09)	5 × 10^–12^
Apolipoprotein B, g/L	692	1.00 (0.01)	344 547	1.03 (0.0004)	–0.03 (–0.05 to –0.01)	4 × 10^–4^
Systolic blood pressure, mm Hg	730	142.30 (0.86)	361 985	144.07 (0.040)	–1.67 (–3.25 to –0.09)	.046
Diastolic blood pressure, mm Hg	730	85.40 (0.50)	361 369	86.30 (0.023)	–0.87 (–1.81 to 0.08)	.058
Glucose, mmol/L	641	5.13 (0.04)	316 827	5.11 (0.002)	0.02 (–0.07 to 0.12)	.054
HbA1_c_, mmol/mol	702	38.01 (0.23)	346 244	38.16 (0.015)	–0.15 (–0.60 to 0.30)	.320
Alanine aminotransferase, U/L	695	23.00 (0.41)	346 097	23.53 (0.024)	–0.51 (–1.53 to 0.50)	.790
Aspartate aminotransferase, U/L	692	25.68 (0.28)	344 948	26.19 (0.018)	–0.49 (–1.26 to 0.26)	.547
Visceral adipose tissue volume, L	38	4.09 (0.39)	19 950	3.77 (0.016)	0.10 (–0.52 to 0.72)	.976
Abdominal subcutaneous adipose tissue volume, L	38	7.33 (0.60)	19 942	6.97 (0.023)	0.55 (–0.40 to 1.51)	.299
Abdominal fat ratio	36	0.49 (0.02)	19 404	0.50 (0.001)	0.00 (–0.03– to 0.03)	.962

These are all LOFTEE high-confidence nonsense, frameshift, and essential splice variants. Medians, quartiles, minimum, and maximum values are available in the Supplementary Table 3. *P* values are based on an inverse normalized variable; nonnormalized *P* values are presented in Supplementary Table 3.

Abbreviations: BMI, body mass index; HbA_1c_, glycated hemoglobin A_1c_; HDL, high-density lipoprotein; LDL, low-density lipoprotein; LoFTEE, Loss of Function Transcript Effect Estimator; PTV, protein-truncating variant.

There was no association with glycated hemoglobin A_1c_ (–0.15 mmol/mol, 95% CI, –0.60 to 0.30 mmol/mol, *P* = .32). Similar results were observed when adjusted for body mass index and stratified by sex (Supplementary Tables 3-6) (7), except waist-to-hip ratio, which was stronger in women (–0.021 [95% CI: –0.028 to –0.015], *P* = 8 × 10^–10^) than men (–0.011 [95% CI, –0.018 to –0.004], *P* = .002). The effects were also consistent when we performed a range of sensitivity analyses, including removing last exon variants that are likely to escape nonsense-mediated decay, stratifying by type of variant and excluding relatively common variants. Supplementary Tables 3 to 7 (7) also provide the results by each of the individual *PLIN1* variants.

### *PLIN1* Protein-truncating Variants Are Associated With Reduced Risk of Myocardial Infarction and Hypertension

We next tested for association with lipodystrophy-associated diseases including all prevalent and incident cases identified in the UK Biobank (Table 3). *PLIN1* PTVs were nominally associated with reduced risk of myocardial infarction (odds ratio [OR] = 0.59; 95% CI, 0.35-0.93, *P* = .02) and hypertension (OR = 0.85; 95% CI, 0.73-0.98, *P* = .03). There was no association with T2D (OR = 0.99; 95% CI, 0.63-1.51, *P* ≥ .999).

**Table 3.** Association of protein-truncating variants in *PLIN1* with lipid- and lipodystrophy-related diseases in the UK Biobank

Trait	PTV carrier frequency	Control frequency	OR (95% CI) exact	*P* (exact)	P (logistic)
Any diabetes	0.094	0.103	0.91 (0.70-1.17)	.502	.540
Type 2 diabetes	0.032	0.032	0.99 (0.63-1.51)	≥ .999	.917
Hypertension	0.495	0.531	0.85 (0.73-0.98)	.028	.025
Coronary artery disease	0.081	0.105	0.76 (0.55-1.02)	.076	.076
Stroke	0.026	0.032	0.81 (0.49-1.29)	.458	.421
Myocardial infarction	0.025	0.041	0.59 (0.35-0.93)	.020	.027
PCOS	0.002	0.003	0.75 (0.02-4.20)	≥ .999	.806

These are all LOFTEE high-confidence nonsense, frameshift, and essential splice variants. *P* (logistic) is an adjusted model accounting for age at baseline, sex, center, and 5 principal components of ancestry. PCOS analyzed in females only.

Abbreviations: LoFTEE, Loss of Function Transcript Effect Estimator; OR, odds ratio; PCOS, polycystic ovary syndrome; PTV, protein-truncating variant.

### Similar Associations of *PLIN1* Protein-truncating Variants Were Observed in a Second, Smaller Independent Cohort

There were 11 *PLIN1* PTVs in 82 individuals in the T2D Knowledge portal. Although there is a reduced statistical power because of the smaller number of individuals in the analyses (maximum N = 19 610 for continuous traits), the results were in the same direction as our UK Biobank analyses. Individuals with *PLIN1* PTVs had increased HDL cholesterol (0.15 mmol/L; 95% CI, 0.02-0.29 mmol/L, *P* = .02), and directionally consistent effects on reduced waist-to-hip ratio (–0.01, *P* = .40), reduced systolic (–0.02, *P* = .41) and diastolic blood pressure (–1.18, *P* = .39), and reduced triglycerides (–0.03, *P* = .78). There was no association with risk of T2D (OR = 0.79, *P* = .48).

A phenome-wide association study suggests no increased risk of adverse outcomes for individuals with *PLIN1* PTVs.

We explored the association of PTVs in *PLIN1* using recently released summary statistics from Regeneron (5). Supplementary Table 8 (7) provides the burden testing results for all *PLIN1* PTVs from these analyses. The results are consistent with our analyses, with the strongest association for PTVs being for increased HDL cholesterol. No disease trait was positively associated with *PLIN1* PTVs after adjusting for multiple testing.

## Discussion

Our large genotype-first approach study suggests that haploinsufficiency of *PLIN1* causes a favorable metabolic profile rather than the adverse metabolic profile of lipodystrophy. We find some evidence of a reduced risk of key disease outcomes including myocardial infarction and hypertension. This suggests that downregulation of *PLIN1* may represent a new target for preventing cardiometabolic disease.

Our large study of 735 people with *PLIN1* PTVs robustly demonstrates that they are associated with favorable lipid profiles (higher HDL and lower triglycerides). These results are similar to our previous small study (n = 6) of cases with *PLIN1* PTVs that were identified as part of the monogenic diabetes testing. Some recent studies of the UK Biobank have also noted that *PLIN1* is associated with higher HDL cholesterol (11, 12), but not all have noted the direction of effect. Recently, Hindy and colleagues identified 35 genes associated with lipid levels (13). *PLIN1* PTVs were associated with higher HDL cholesterol (3.9 mg/dL, *P* = 1 × 10^–5^) and nominally with reduced triglycerides (–7%, *P* = .02). This included an overlapping 40 586 individuals from the first release of UK Biobank exome data. Our data, including an additional 380 000 UK Biobank individuals, now provides conclusive evidence that *PLIN1* haploinsufficiency is associated with a favorable metabolic profile. The lack of selection bias and uniform assessment of lipid profiles means that our results are likely to represent the true effect of *PLIN1* PTVs in humans. Our results are in contrast to the previous 3 family-based studies showing that *PLIN1* frameshift variants have lower HDL, severely raised triglycerides, and partial lipodystrophy (1-3). However, these individuals had frameshifts (p.Val398Glyfs*166, p.Tyr401Leufs*165, p.Pro403Argfs*164, p.Leu404Alafs*158, and p.Pro439 Valfs*125) at the end of the penultimate exon or in the last exon, which create a PLIN1 protein with an altered 150–amino acid C-terminus. There were only 13 individuals with frameshift variants in the last exon in our study and none that resembled the published pathogenic variants. These 13 individuals have decreased HDL cholesterol levels (–0.26 mmol/L; 95% CI, 0.73 to 0.98 mmol/L, *P* = .01), but not significantly increased triglycerides (0.26 mmol/L; 95% CI, –0.33 to 0.85 mmol/L, *P* = .30).

Our results suggest that *PLIN1* PTVs may cause some reduction in risk of cardiovascular disease and blood pressure. Cardiovascular disease is the leading cause of death in the world, and there is a real need for new therapeutic drugs to address this global disease (14). Our results show that *PLIN1* PTVs are associated with a 1 to 2 mm Hg reduction in blood pressure, lower diagnosis of hypertension, and lower rate of myocardial infarction. This is consistent with the recent results from an exome-wide, phenome-wide association study of the 454 757 UK Biobank exomes from Backman et al (5) that found a reduced risk of myocardial infarction (OR = 0.55, *P* = .008) and hypertension (OR = 0.71, *P* = .05) and with a stricter definition of coronary artery disease (OR = 0.65, *P* = .008). However, the opposite result was observed in rodents in which mice lacking *PLIN1* showed increased blood pressure (15). This may be due to the well-known lack of similarities of human and mouse hearts and physiology (16). The possible underlying mechanism of lower cardiovascular disease seen in individuals with PLIN1 PTVs is not known but it could be secondary to lower blood pressure in combination with a favorable lipid profile seen in these individuals. Further, even larger studies are needed to validate this association because the associations with blood pressure and hypertension in particular are weak.

We show that the phenotype of individuals with *PLIN1* haploinsufficiency is inconsistent with the presence of clinical partial lipodystrophy (low HDL, high triglycerides, adverse metabolic disease). These mutation-specific effects are a feature of several lipodystrophy genes. For example, we have shown that only a specific in-frame deletion that affects the polymerase domain of *POLD1* causes a lipodystrophy syndrome (17), whereas mutations in the exonuclease domain have been shown to cause colorectal cancer (18). Another example is *LMNA*, in which only specific missense mutations cause lipodystrophy and other variants cause distinct phenotypes including progeria, whereas haploinsufficiency causes cardiomyopathy (19).

Our study cannot form any conclusions about protein-extending frameshift variants. Only 7 of the variants occurred in the last exon, and none of the previously reported lipodystrophy variants are present in the 454 757 UK Biobank exomes assayed here. Our study provides further evidence, however, that only specific frameshift variants that extend the translated protein cause severe lipodystrophy. This means that novel PTVs of *PLIN1* should not be reported back to patients as a cause of their lipodystrophy.

In contrast to the originally reported monogenic lipodystrophy syndrome, *PLIN1* haploinsufficiency causes a favorable metabolic profile and may protect against cardiovascular disease.

## Data Availability

Some or all data generated or analyzed during this study are included in this published article or in the data repositories listed in “References.”

## Abbreviations

HDL: high-density lipoprotein
ICD: International Classification of Diseases
OR: odds ratio
PTV: protein-truncating variant
T2D: type 2 diabetes
